# Evaluating Non-Conventional Chitosan Sources for Controlled Release of Risperidone

**DOI:** 10.3390/polym14071355

**Published:** 2022-03-26

**Authors:** Sara Garcinuño, Inmaculada Aranaz, Concepción Civera, Concepción Arias, Niuris Acosta

**Affiliations:** 1 Pluridisciplinar Institute, Complutense University, 28040 Madrid, Spain; garcinunosara@gmail.com; 2 Department of Chemistry in Pharmaceutical Science, Pharmacy Faculty, Complutense University, 28040 Madrid, Spain; mccivera@ucm.es (C.C.); carias@ucm.es (C.A.)

**Keywords:** risperidone, injectable formulation, xerogels, hydrogels, β-chitin, controlled release, chitosan

## Abstract

In this work, two chitosan samples from cuttlebone and squid pen are produced and characterized. We studied the formation of thermoresponsive hydrogels with β-glycerol phosphate and found proper formulations that form the hydrogels at 37 °C. Gel formation depended on the chitosan source being possible to produce the thermoresponsive hydrogels at chitosan concentration of 1% with cuttlebone chitosan but 1.5% was needed for squid pen. For the first time, these non-commercial chitosan sources have been used in combination with β-glycerol phosphate to prepare risperidone formulations for controlled drug delivery. Three types of formulations for risperidone-controlled release have been developed, in-situ gelling formulations, hydrogels and xerogels. The release profiles show that in-situ gelling formulations and particularly hydrogels allow an extended control release of risperidone while xerogels are not appropriate formulations for this end since risperidone was completely released in 48 h.

## 1. Introduction

Chitin is a copolymer composed mainly of β-(1-4) linked *N*-acetylglucosamine and glucosamine residues, the latter in a very low proportion. Chitin can be found in the exoskeleton of arthropods such as crustaceans and insects, in mollusks such as cephalopods and in algal and fungal cell walls [1].

Two mainly crystalline forms can be found in chitin: α- and β-chitins [2]. The most abundant and easily accessible form is α-chitin, where the molecules are aligned in an antiparallel fashion with strong intermolecular hydrogen bonding between chains. This is the type of chitin isolated from crustaceans and insects. In β-chitin, the molecules are packed in a parallel arrangement, leading to weaker intermolecular forces. This chitin is isolated from cephalopods such as squid and cuttlefish. Chitosan, the deacetylated derivative of chitin only exits in the wall cell of fungi and it is usually produced by chemical deacetylation of chitosan. Due to the large presence of glucosamine units in its chain, chitosan is soluble in acidic aqueous solutions and therefore has more interest in application than chitin which is a highly insoluble polymer. Chitosan is biodegradable, non-toxic, biocompatible and has very interesting properties such as antimicrobial, antifungal or antioxidant among others so it is a very promising polymer for biomedical applications [3]. Commercial chitin and chitosan come from the marine food industry from crustacean shells. The use of cephalopods to produce chitosan for biomedical applications is less explored since it is not the typical commercial sample.

Chitosan is widely used in biomedical and pharmaceutical applications, not only due to its biological properties but also due to its technological properties. Chitosan have been used successfully to obtain films, micro and nanoparticles, scaffolds, hydrogels and different drug delivery systems [4,5,6,7].

Of particular interest are chitosan thermoresponsive gels produced with β-glycerolphosphate [8,9]. In this formulation, chitosan dissolved in acidic media is mixed with β-glycerolphosphate at 4 °C. In these conditions, although the pH of the solution is higher than chitosan pKa the polymer remains in its soluble state. Once the temperature has raised, a hydrogel is produced. The temperature at which this transition occurs depends on the polymer and β-glycerolphosphate concentrations and as well as chitosan characteristics. These chitosan thermoresponsive hydrogels have been proposed in many drug delivery applications [9]. Most of the published studies on the application of thermoresposive chitosan gels with β-glycerol phosphate are based on commercially available chitosan derived from crab or shrimp, and fewer studies have investigated the potential application of β chitosan. Squid pen chitosan thermoresponsive hydrogels have been explored in bone tissue engineering [10] and cartilage tissue engineering [11]. The effect of silver nanoparticles in gelation time using squid pen chitosan has also been studied [12].

Risperidone (RSP) is an atypical antipsychotic which acts by blocking the serotonin (5HT2A) and dopamine (D2) receptors [13]. This drug is widely used to treat schizophrenia and bipolar disorder. It is a poorly soluble drug in aqueous media under physiological conditions (aqueous solubility at 25 °C and 37 °C is 2.8 and 5 µg/mL, respectively) [14]. Moreover, adherence to antipsychotic medications is a major challenge in schizophrenia. Therefore, its formulation is critical for an appropriate drug release pattern. Different risperidone formulations for extended-release have been described, for instance, RSP has been formulated in polymeric PEG-PGLA microparticles has [15], in fast dilution tablets [16], in pegylated proteinoid nanocapsules [16], in PLGA-microplates [17] or orodispersible films [18]. Chitosan has been used in the formulation of RSP drug delivery system in the form of nanoparticles [19] and mucoadhesive nanoemulsions [20].

RSP delivery formulations have been designed for different administration routes such as transdermal patches [21], oral transmucosal delivery [22], oral delivery [23,24], injectable formulationsri [25,26] or intranasal delivery [19].

The aim of this work is to evaluate the use of non-commercial chitosan samples in the formulation of risperidone, as hydrophobic drug model, by taking advance of the thermal gelling properties of chitosan in the presence of β-glycerolphosphate. We prepared and evaluated three different formulations, hydrogels, in-situ gelling formulation for injectable applications and xerogels (dehydrated hydrogels by freeze-drying).

## 2. Materials and Methods

### 2.1. Materials

Cuttlebone from common cuttlefish (*Sepia officinalis*) was collected on the Valencian coast in May 2019 and the squid pen was obtained from waste from the fishing industry (Mercado de Villa de Vallecas, Madrid, May 2019). *N*-acetyl-d-glucosamine, β-glycerol phosphate (βGP), and phosphate-buffered saline was purchased from Sigma-Aldrich (St. Louis, MO, USA) Risperidone (RSP) was kindly donated by InFiQuS, S L. Other chemicals were of analytical grade and supplied from Panreac (Barcelona, Spain).

### 2.2. Methods

#### 2.2.1. β-Chitosan Isolation

β-chitosan was isolated from cuttlebone and squid pen in several steps. In the first place, the raw material was thoroughly washed with water to remove sand. After, the samples were boiled in water for 1 h to remove organic matter and after being air-dried the samples were crushed with a coffee grinder (Sinbo SCM 2931) to a particle size of around 5 mm. Demineralization was carried out by means of an acid hydrolysis process adapting the methodology from Arrouze et al. [27]. Samples were put in contact with 1 N HCl for two hours while stirring at room temperature. The chosen ratio between the samples and the HCl was 1:4 (*w*/*v*) for the cuttlebone and 1:8 (*w*/*v*) for the squid pen. During the process, the pH of the solution was periodically checked to be acid; if not, the neutralized acid was removed, and a new solution was added. After two hours, the samples were filtered and successively washed with distilled water until the pH of the washing water was neutral. Finally, they were collected and left to dry overnight at 45 °C in an oven (Vaciotem, Selecta).

Deproteinization was carried out by means of an alkaline hydrolysis process by modifying the protocol described by Azourre et al. [27]. Briefly, the sample was put in contact with 4% NaOH (80 °C) for two hours at room temperature under agitation, with a ratio between the sample and NaOH of 1:15 (*p*/*v*) for the cuttlebone sample and 1:10 (*w*/*v*) for the squid pen. The deproteinized samples were washed successively with acidified water (200 μL of lactic acid for every 5 L of distilled water) until the pH of the washing water was neutralized. The samples were then collected and allowed to dry at 45 °C overnight. This sample was β-chitin. The isolated β-chitin was weighed (precise analytical balance S-300) to calculate the isolation yield and the deacetylation process was carried out to obtain chitosan. This process (consisting of alkaline hydrolysis) was carried out with NaOH at 70% (*p*/*v*) for four hours at 100 °C under agitation, with a ratio between chitin and NaOH of 1:10 (*p*/*v*) [28]. The samples were successively washed with acidified water until the pH of the washing waters was neutral. Finally, the deacetylated samples were left to dry at 45 °C overnight.

#### 2.2.2. Polymer Characterization

Chitosan acetylation degree was determined by the UV-VIS spectrophotometric method of the first derivative [29]. Chitosan viscosity and molecular weight were determined by viscosimetry. The viscosity measurements were performed using a Ubbelohde capillary viscometer (525–20 capillary, West Conshohocken, PA, USA) at 25.0 ± 0.1 °C. The used solvent was 0.3 M AcOH/0.2 M AcNa. The average viscosity molar mass was calculated from the Mark Houwink law (Equation (1)):

[*η*] = *K* × M_v_*^α^* (1)

where *η* is the intrinsic viscosity, *K* and *α* are two constants that depend on the nature of the polymer, and M_v_ is the average viscous molecular weight. Under our experimental conditions *K* = 0.00076 and *α* = 0.76 when the intrinsic viscosity is expressed in dL/g [30].

Chitosan moisture content was determined gravimetrically in triplicate. The samples were weighed and dried at 105 °C under vacuum for 3 h to remove moisture. The percentage of moisture was determined using Equation (2).

(2) $$\mathrm{Humidity}\;(\%)=\frac{w_{h}-w_{d}}{w_{h}}\times 100$$

where *w_h_* is the initial weight of the sample and *w_d_* is the weight of the sample once dry.

The inorganic matter content (Ash content) in chitosan samples was determined in triplicate. The samples were incinerated at 800 °C for 8 h and the residual matter was weighed.

(3) $$\mathrm{Ashes}\;(\%)=\frac{w_{2}}{w_{1}}\times 100$$

where *w*_1_ is the weight of the dehydrated sample and *w*_2_ is the weight of the sample once incinerated.

FTIR-ATR characterization was carried out using a Cary 630 FTIR spectrometer (Agilent Technologies, Santa Clara, CA, USA). The determination was carried out between 650 and 4000 cm^−1^ at a resolution of 4 cm^−1^ for 32 scans.

XRD patterns were determined in a PANalytical model X’Pert MPD multi-purpose diffractometer with copper radiation. The samples were measured at an angular range from 5° to 60° 2θ angle. The crystallinity index (CrI) was determined according to Equation (4) [31]:

(4) $$\mathrm{CrI}\;(\%)=\frac{I110-Iam}{I110}\times 100$$

Being *I*110 the maximum intensity of the reflection at 2θ = 20° and *Iam* the minimum intensity of the diffraction in the amorphous region at 2θ = 16°.

#### 2.2.3. Preparation of Hydrogels

The gelation capacity of the obtained chitosans was determined using βGP [4]. Chitosan (0.5%, 1% and 1.5% *w*/*v*) was dissolved in 0.1 M acetic acid and βGP in distilled water (5%, 10% and 15% *w*/*v*). In an ice bath, 750 μL of the chitosan solution were mixed with 250 μL of the βGP solution, added dropwise and with vortex agitation (Heidolph Reax top, Schwabach, Germany). Once the two components were mixed, they were left at room temperature for five minutes and then introduced into a thermoblock (tembloc, Selecta, Barcelona, Spain) previously equilibrated at 37 °C. Gelation time was determined by the test tube inverting method, the fluidity of the samples was checked every 60 s by inverting the tube. The time at which flow stopped was taken as the gelation time and the values were recorded.

#### 2.2.4. Risperidone Load Formulation

To produce injectable formulations a known weight of risperidone was dissolved in AcOH and added to chitosan solution (drug content was 10% weight regard to polymer), then the βGP dissolved in cold water was added drop by drop into the polymer-drug solution in an ice bath. After 5 min at room temperature, the mixture was introduced into a dialysis membrane in a PBS bath at 37 °C.

Risperidone hydrogels were produced by preparing a polymer-drug-βGP solution as previously described. The mixture was kept at room temperature for 5 min and the sample was incubated at 37 °C for 5 h to produce a robust hydrogel.

Xerogels were prepared by introducing the chitosan-drug-βGP samples into 1 mL syringe. The syringe was incubated at 37 °C for 5 h to form a hydrogel and then frozen at −80 °C. The syringe was cut into pieces of around 1 cm and lyophilized. Table 1 summarizes the characteristics of each formulation.

Chitosan concentration in the formulations was 1% in the case of squid pen chitosan and 1.5% in the case of cuttlebone chitosan. This selection was carried out considering previous results of chitosan gelation in the presence of β-GP as describe in results section.

The morphology of the lyophilized hydrogels was studied by scanning electron microscopy (SEM). To do this, in the microscopy CAI of the UCM, the samples adhered to sample holders with double-sided tape were metallized with gold (Quorum Q150R S metallizer, Lewis, UK) for 180 s at 30 mA. Once metallized, the samples were observed in a scanning electron microscope (JEOL JSM 6335F, Tokyo, Japan) at an electron acceleration voltage of 20 kV.

#### 2.2.5. Risperidone Release Profile Studies

The RSP release kinetic was determined under physiological conditions (PBS, pH 7.4, 37 °C) in a volume of 10 mL. Samples of 500 μL of release medium were replenished by a fresh medium. This study was carried out in triplicate and quadruplicate.

The concentration of RSP was determined by UV spectrophotometry at 278 nm. A calibration curve was obtained to quantify the amount of risperidone released over time. Dilutions of known drug concentration (from 1.5 μg/mL to 50 μg/mL) were prepared and their absorbance at 278 nm was determined (Specord 205, Analytik GmbH, Jena, Germany).

The risperidone release kinetics was evaluated by fitting the experimental data obtained from the release assay through several mathematical models (zero-order, first-order, Higuchi, Korsmeyer-Peppas and Weibull), by employing DDSolver add-in program from the Microsoft Excel application.

## 3. Results

### 3.1. Chitosan Characterisation

**Figure 1:**
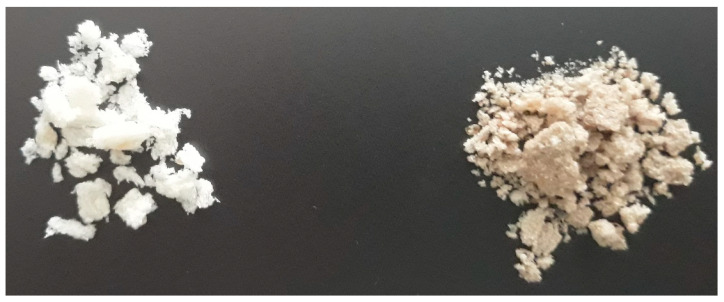
Optical visualization of chitosan samples. Squid pen chitosan (**left**) and cuttlebone chitosan (**right**).

In this work, we isolated β-chitin from two different sources, cuttlebone and squid pen, and these chitins were deacetylated to produce chitosan by a heterogeneous chemical procedure [32]. Due to differences in the raw material, the procedure was adapted for each sample. To cuttlebone demineralization, a stronger procedure was carried out due to its higher mineral content [33]. In deproteinization, the ratio sample NaOH solution was also increased for the cuttlebone sample since this sample completely absorbed the basic solution in the ratio 1:10. Chitin yields for cuttlebone and squid pen were around 4% and 35%. To deacetylate β-chitin to produce chitosan, the same reaction conditions were used. Chitosan from squid pen was a white powder while chitosan from cuttlebone has a light brown colour (Figure 1). Squid pen has very low mineral content and chitosan yield was higher than the one observed for cuttlebone (25.38 vs. 1.64 from raw material: 71 vs. 50% from β-chitin, respectively).

The main physicochemical characteristics of both chitosan samples are shown in Table 2. Squid pen showed more purity than cuttlebone chitosan since its residual inorganic material was lower than 0.5%. Small differences were determined regarding their acetylation degree while squid pen chitosan exhibited larger molecular weight and it was clearly more viscous.

**Figure 2:**
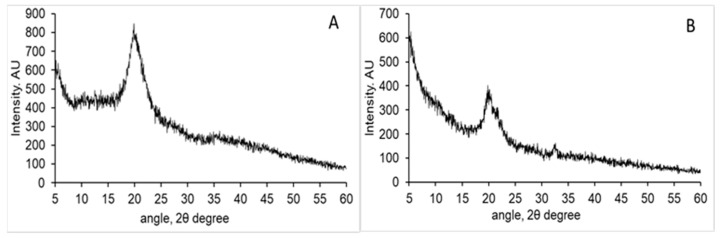
XRD pattern of squid pen chitosan (**A**) and cuttlebone chitosan (**B**).

XRD patterns of both samples are shown in Figure 2, the typical pattern of a semicrystalline polymer was observed with a strong reflection at 20° 2θ angle which corresponds to 110 planes [34]. In chitosan isolated from squid pen a broad peak around 20° 2θ angle appears while such reflection is not observed in cuttlebone chitosan. Crystallinity degree was determined to be around 50% in both samples.

**Figure 3:**
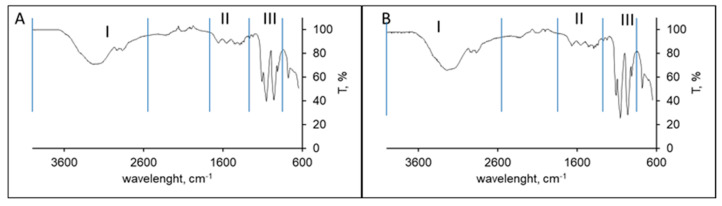
FTIR-ATR spectra of cuttlebone chitosan (**A**) and squid pen chitosan (**B**). Regions I, II and III are indicated in the figure.

The ATR-FTIR spectra of both chitosan samples were quite similar as seen in Figure 3. Both spectra exhibited three main spectral regions. A first region appears as a broad asymmetric band between 3400 and 2500 cm^−1^ due to the CH stretching modes at around 2927 and 2867 cm^−1^ and, at higher wavenumbers (around 3229 cm^−1^), bands corresponding to the overlapped O-H and N-H stretching vibrations; the second region appears between 1700 and 1200 cm^−1^ and it is characteristic of the amide groups (amide I at 1650 cm^−1^), finally, the third region appears as a strong absorption region between 800 and 1200 cm^−1^ characteristic of the chitosan saccharide structure In particular, bands at 1154 and 1074 cm^−1^ are attributed to C-O-C stretching vibration modes [35].

### 3.2. Chitosan Thermoresponsive Gels

Appropriate gelation time of the hydrogel is of much importance for clinical or biomedical applications. Large gelation times produced the diffusion of the gel inside the injection site and poorly drug-controlled release. The formation of chitosan gels using different chitosan and βGP concentrations at 37 °C was evaluated by the inversion tube methodology. At low polymer concentration (0.5%) no gel formation was observed no matter the chitosan sample analyzed. Squid pen produced thermoresponsive gels only at high concentration (1.5% *w*/*v*), while cuttlebone chitosan was able to produce the thermoresponsive gels at 1% *w*/*v*. In both polymers, gelation time was reduced as the βGP concentration increased (Table 3). Chitosan gelation also depends on the temperature, when samples were assayed at higher temperatures (40 and 45 °C) a slight reduction of the gelation time was observed but still, no gelation occurred on those formulations that did not form gels at 37 °C (Data not shown).

### 3.3. Chitosan-Based Risperidone Release Formulations 

**Figure 4:**
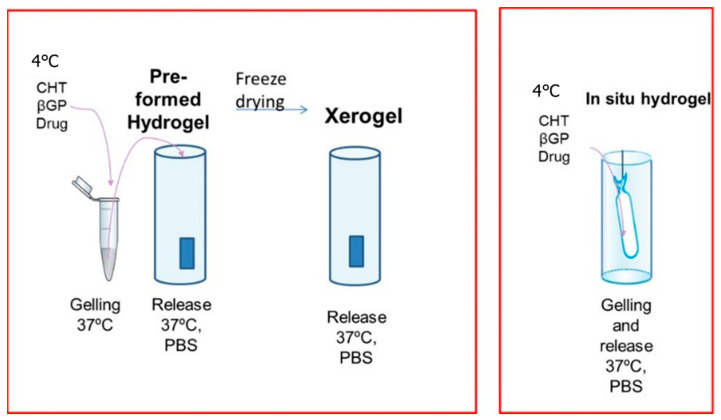
Risperidone formulations.

Taking advance of the gelling properties of chitosan-β glycerolphosphate mixtures, we prepared three types of formulations for risperidone release (Figure 4). An in-situ gelling formulation for injectable systems, a hydrogel and a xerogel consisted of lyophilized hydrogels. To produce these formulations a concentration of 1% and 1.5% of cuttlebone and squid pen chitosan was selected, respectively. Gelation time was fixed to 5 h and the concentration of βGP was 10%, in this way we were sure that hydrogels were formed without highly increasing formulation osmolarity.

**Figure 5:**
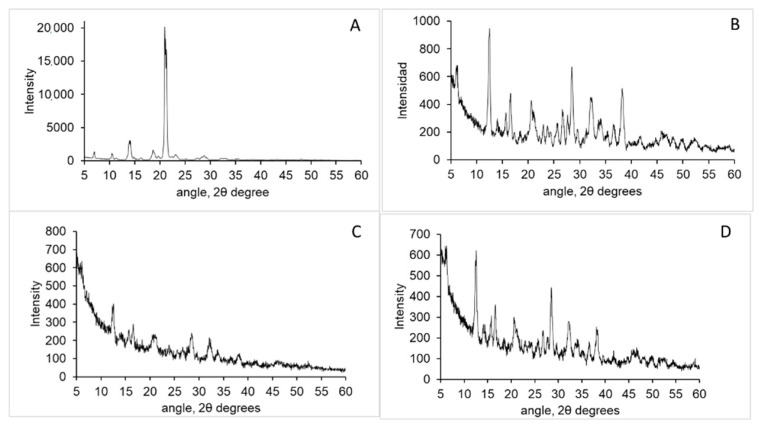
XRD patterns of Risperidone (**A**), glycerolphosphate (**B**), xerogel from cuttlebone chitosan (**C**) and xerogel from squid pen chitosan (**D**).

RPS is a crystalline drug with a main reflection at 21° 2θ angle and small reflections at 7°, 10.5°, 14°, 19° and 28° 2θ angle (Figure 5A). Also, βGP is a crystalline salt whose main reflections appear at 12.5°, 16.7°, 28.5°, 32.4° and 38.2° (Figure 5B). The XRD spectra of xerogels showed the typical reflections of βGP which mainly are overlapped with those of risperidone (Figure 5C,D). From the spectra was not possible to determine the reflections of risperidone on the samples. This can be due to several factors: (i) RSP reflections are overlapped with βGP and RSP intensity at 21° 2θ is much lower in intensity than βGP reflections. (ii) RSP maybe in amorphous state in the formulation and therefore no reflections are observed or (iii). RSP crystal size is very small and therefore no reflections are observed. 

**Figure 6:**
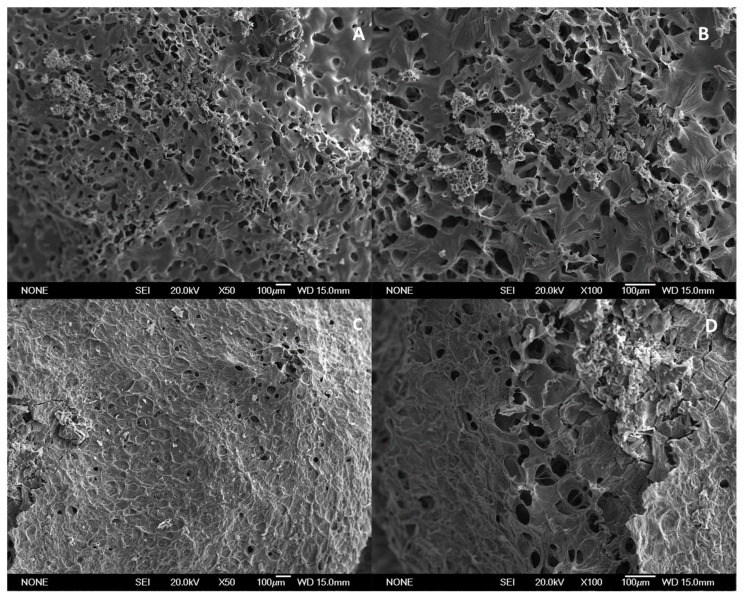
SEM images of xerogels from cuttlebone (**A**,**B**) and squid pen chitosan (**C**,**D**).

The morphological features of the xerogels are shown in Figure 6. Porous xerogels were prepared by using ice as a template in a green methodology [36]. This methodology avoids the use of toxic solvents and therefore is very appropriate for biomedical applications. The images show that cuttlebone chitosan xerogels have a more open porous structure than the sample from squid pen.

**Figure 7:**
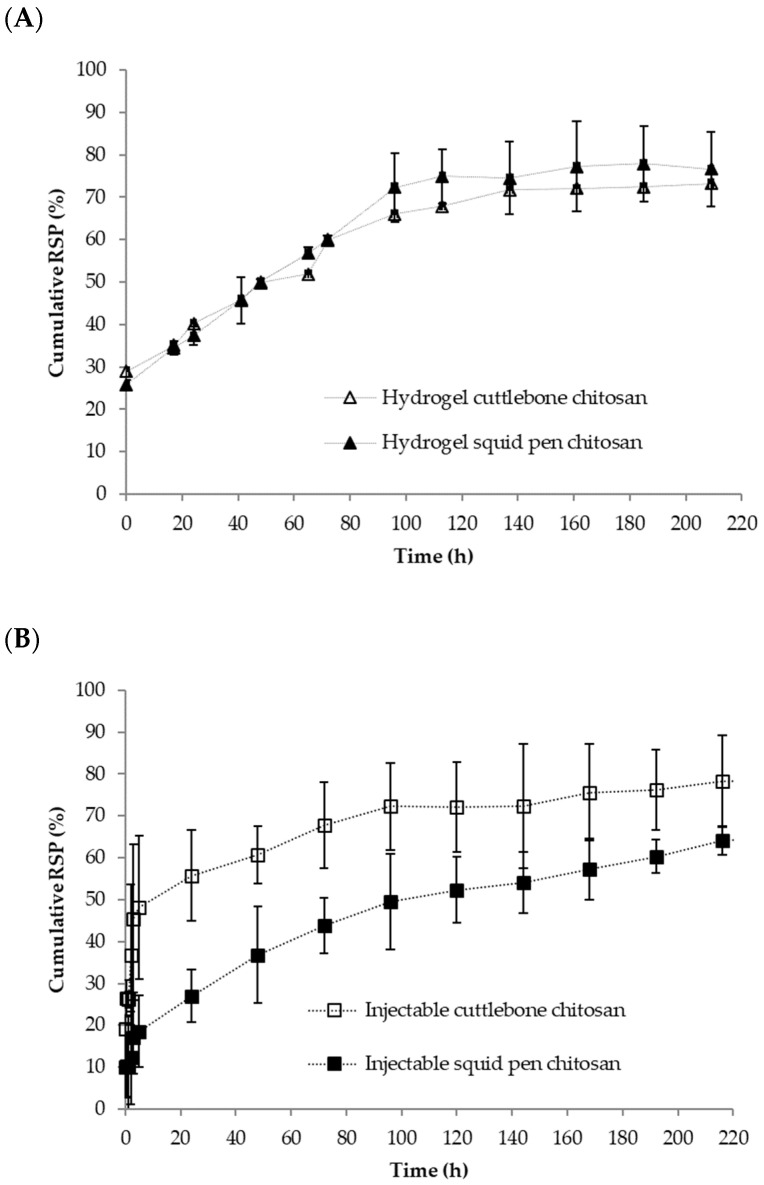

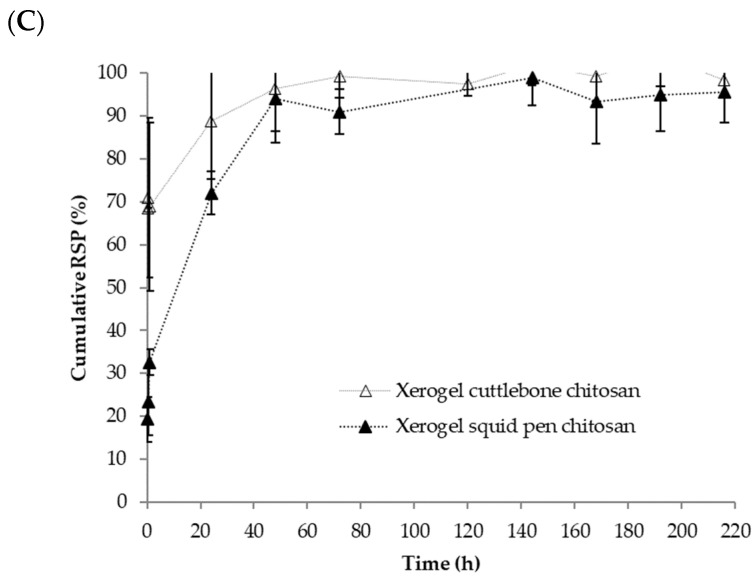
Cumulative release of risperidone from different formulations: (**A**) Hydrogels, (**B**) Injectables, (**C**) Xerogels.

The release profiles for the three formulations over 8 days are shown in Figure 7. As can be seen for the hydrogel formed in-situ (injectable formulation, Figure 7A) the cuttlebone chitosan hydrogel showed an initial burst RSP release of 20% followed by a sustained release, reaching a maximum release percentage of 80% after 8 days. The squid pen chitosan hydrogel presented an overall lower release, with an initial burst release of 10% followed by a sustained release that reach a value of 65% at 8 days. The differences in the initial release could be explained by the different gelation times of both formulations. Cuttlebone formulation gelled in 10 min while squid pen just needs 5 min to gelation. RSP release starts whereas chitosan is gelling, the larger the gelation time the less retention is expected which is in good agreement with our results.

The SPR release patterns from the hydrogels were quite similar (Figure 7B), initially, around 25% of the drug was released followed by a sustained release over time with a maximum release close to 75% after eight days. Samples were also analyzed at 28 days and around 83% of the drug was released at this time indicating that this formulation permits an extended release of SPR. In this case, since chitosan is completely forming a hydrogel when the formulation is put in contact with the release media the effect of gelling time is neglected.

The main differences were observed in the case of xerogels. The squid pen xerogels presented an initial release of 20% and a sustained release over time in the following two days, reaching the maximum release in 48 h. In the case of the cuttlebone xerogels, the initial release was 70% and in 24 h practically all the risperidone was released.

Furthermore, to study the sustained release mechanism and kinetics of the formulations the release profiles were fitted to different models (Table 4). For hydrogels and xerogels, the suitability of different common empirical (zero-order, first-order, and Higuchi), and semi-empirical (Korsmeyer-Peppas) models, and statistical (Weibull), models to describe the drug release profile were tested through non-linear least-square curve fitting, by employing DDSolver. Further, instead of the widely used transformed linear fit method, direct fitting was used in the paper to avoid any form of truncation and transformation errors (Table 4). The Akaike information criterion (AIC) and the correlation coefficient R^2^, were used to determine the best model.

Release data were firstly adjusted to Korsmeyer-Peppas model since it provides descriptive information on both release kinetics and mechanism of drug release. It should be underlined that the *K* and *n* were determined in the range of Mt/M ∞ 0–60%.

As observed in Table 4, all gels formulations (hydrogels and in-situ formulations) fit well to Korsmeyer-Peppas model. Best results were obtained for squid pen formulation with R^2^ almost 0.99 and the lower value of AIC was for the injectable formulation. For the hydrogel produced with squid pen chitosan the exponent *n* was 0.45 so the diffusional release could be assumed as Fickian diffusion. The other formulations have exponents lower than 0.5, so the diffusional release was assumed to be quasi-Fickian diffusion. The parameter *K* indicated the constant drug transport [37], that can be seen intuitively as directly proportional to the drug release kinetics. So faster drug release was obtained for cuttlebone chitosan hydrogel. On a contrary, lower value of *K* indicated a low transport kinetic, thus injectable formulation with chitosan from squid pen showed the slowest release kinetics.

To further understand the results obtained, Higuchi equation was selected. Main conditions to apply this model are a high initial excess of drug and a no swelling, no ointment base dissolution [38,39]. Our data did not fit well to this model, best result was obtained for squid pen chitosan hydrogel formulation with value of *K_H_* of 0.13 h^½^ and R^2^ 0.936.

The Weibull model gave successful fittings for almost the entire release curve (only high values of 648 and 672 h were excluded). The exponent of time β of the Weibull function is linearly related to the exponent *n* of the power law derived from the analysis of the first 60% of the release curves. The value of the exponent β is an indicator of the mechanism of transport of a drug through the polymer matrix [40]. All formulations gave b ≤ 0.75 indicate Fickian diffusion in agreement with Korsmeyer-Peppas model fit. Interestingly, squid pen chitosan hydrogel gave the better fit and the injectable cuttlebone formulation exhibited a bigger value for b and bigger R^2^. The fitting of gels release data to first order kinetic gave low R^2^, although different data of the curve were selected. Although, pseudo zero-order kinetic is traditionally used for the kinetic of carbohydrates our data did not fit to this model [41]. It is worth noting that it was not possible to fit release data of xerogels to any of the proposed models.

## 4. Discussion

Chitin and chitosan yields depend on the origin of the raw material and the procedure for chitin isolation and chitosan production. For instance, chitin yields of 70% have been described for krill, 60% for crab and 30% for *S. kobiensis* cuttlebone [42] and 20% for *S. pharaonis* cuttlebone [43]. This variety is due not only to the origin of the sample, but it is also related to how the sample has been processed. Arrouze et al. separated the cuttlebone (*Sepia officinalis*) in two parts (shell and thin layer) previously to chitin isolation finding than in the first sample chitin content was 11% while in the second one was 40%. They also found a chitin yield of 42% for squid pen chitosan [27]. These values are higher than those reported in this work but since different protocols were followed to obtain the chitins is difficult to establish the origin of the difference.

Chitosan deacetylation degree and chitosan molecular weight are parameters with strong effect on chitin and chitosan technological and biological properties [44]. In this work we isolated samples with similar deacetylation degree but the differences in molecular weight were remarkable. This difference can be explained considering that due to the low mineral content in squid pen, the extraction conditions for this sample is milder than the one used for cuttlebone avoiding the molar mass reduction from polymer depolymerization by acid hydrolysis. Moreover, source can also be linked to this difference since other authors have also observed higher mass in squid pen chitosan when compared to cuttlebone chitosan isolated under similar conditions [27]. Thermogelling chitosan/glycerolphosphate solutions were first described by Chenite et al. [8]. When chitosan solutions are mixed with βGP at 4 °C although the pH is over chitosan pKa, the polymer maintains its solubility and undergoes gelation while heating the solutions from 4 to 37 °C and above. In this paper, we studied the gelation behaviour of squid pen and cuttlebone chitosan with different polymer and βGP concentrations. The gelation time of both samples was affected by the molecular weight. Samples with the same chitosan concentration have lower gelation time when the sample of higher molecular weight was evaluated. Kolawole et al. [45] have found that the gelation time measured by tube inversion methodology was reduced when a higher molecular weight chitosan sample was tested but no relevant differences in the gelation time were observed when measured by rheology studies. Zhou et al. [34] studied chitosan concentration, chitosan molecular weight and chitosan deacetylation degree on the gelling process. An increase in chitosan Mw or concentration produces an increase in the chitosan-βGP solution viscosities due to the formation of the gels. For a chitosan sample of high molecular weight and a concentration of 2%, gelation time of around 10 min was observed with is in the range of data presented in this work indicating that chitosan source has not strong effect in this parameter.

Ice template is a well-known methodology to induce porosity in freeze-dried scaffolds [46]. Previous studies have shown that the increase of polymer concentration renders to small porous size due to the difficulty to generate large ice crystals. Apart from polymer concentration, which is not highly different in our formulations, we consider that polymer viscosity will also have a relevant role in ice crystal formation. It has been previously reported that high molecular weight chitosan renders a more viscous chitosan-βGP solution [34] and viscosity is a parameter that can hindered ice crystal growth rendering to smaller porous size [46] in good agreement with our results. The differences in porosity may explain the different release behaviour observed in the xerogels. Cuttlebone chitosan xerogels presented a more open structure and therefore the RSP is more accessible to the release media in this sample. Therefore, SPR release is faster from cuttlebone chitosan xerogels. Comparing the release profile of xerogels with hydrogels and in-situ hydrogels we observed a more sustainable release when compared to xerogels. In the case of in-situ hydrogels, some initial release was observed being more relevant in the case of cuttlebone. The differences in the initial release could be explained by the different gelation times of both formulations. Cuttlebone formulation gelled in 10 min while squid pen just needed 5 min to gel. In these in-situ formulations, RSP release starts whereas chitosan is gelling, the larger the gelation time the less retention is expected which is in good agreement with our results. In the case of the hydrogels, initial release was also observed in both formulations. In this case, since chitosan is completely forming a hydrogel when the formulation is put in contact with the release media the effect of gelation time is neglected, and this initial release may be ascribed to the presence of surface drug which can be easily dissolved. After this initial release both formulations showed a similar slow-release pattern. To gain more insight into our formulations, the release patterns were adjusted to different models. Korsmeyer-Peppas model was proven to be the most suitable to analyse hydrogel formulation obtained in this study, providing important information on the release mechanism showing quasi-Fickian diffusion. Ebrahimi et al. have released risperidone from preformed hydrogels of silk fibroin HCl/acetone. They obtained similar results to us, but with a somewhat faster release, obtaining 100% release at 8 days. Extended release was observed when methanol silk fibroin hydrogels were tested [47]. Polymer blends of poly(ε-caprolactone)/poly (propylene glutarate) were investigated for transdermal delivery of RSP in phosphate buffer at pH 7.4 [21]. Release values were observed between 10–40% at 120 h which is a slower release that the one observed in our formulations. The release of these formulations also followed the Korsmeyer–Peppas model. Risperidone controlled release microspheres based on Poly (Lactic Acid)-Poly (Propylene Adipate) for injectable delivery have been evaluated for extended RSP release. These formulations showed different release pattern depending on microspheres composition but in general less than 50% of RSP was released within the first 6 days [25]. In view of these results, we observed that chitosan-βGP formulations exhibit a release pattern comparable to other published formulations.

## 5. Conclusions

In this paper, we evaluated two non-commercial chitosan sources squid pen and cuttlebone chitosan to formulate risperidone drug delivery systems. Chitosan isolation procedure have a strong relevance on the polymer molecular weight and viscosity. These properties are related to the thermosensitive response of the polymers in the presence of βGP and affect the gelation time of the polymeric solutions. To study the control release of risperidone from chitosan-βGP solutions we produced three different formulations: hydrogel, xerogel and injectable (in-situ) formulations. Release patterns of each formulation are related to their intrinsic properties. Xerogels with an open porous structure exhibited a fast release and more than 70% of the drug is released in the first 24 h discarding these formulations for extended drug release. On the contrary, hydrogel and injectables hydrogels have longer release, up to eight-days. In particular, RSP hydrogel with chitosan from squid pen showed the slowest release kinetics and almost a Fickian behavior, indicating that this formulation permits an extended release of risperidone. Due to our results we consider that these formulations could be used as injectable (in situ gelling formulation) or used in transdermal or implants (hydrogels).

## Figures and Tables

**Table 1:** Risperidone formulations.

Formulation	Gelation Time, (h)	Site of Gelation	Freeze-Drying
Injectable	0	Dialysis Membrane ^1^	No
Hydrogel	5	Eppendorf tube	No
Xerogel	5	Syringe 1 mL	Yes

^1^ Dialysis membrane of 7000 Da.

**Table 2:** Chitosan Characterization.

Property	Cuttlebone Chitosan	Squid Pen Chitosan
Humidity (%)	7.76 ± 0.06	5.50 ± 0.14
Ash content (%)	1.65 ± 0.01	0.22 ± 0.01
Acetylation Degree (%)	7.53 ± 0.01	5.93 ± 0.01
Intrinsic viscosity (g/dL)	1.20 ± 0.15	6.63 ± 0.11
M_v_ (kDa)	16 ± 3	153 ± 3

**Table 3:** Thermal gelation of chitosan solutions with beta glycerol phosphate at 37 °C.

% βGP	% Squid Pen Chitosan			% Cuttlebone Chitosan		
	0.5%	1%	1.5%	0.5%	1%	1.5%
5%	-	-	10 min	-	-	30 min
10%	-	-	5 min	-	10 min	10 min
15%	-	-	2 min	-	5 min	5 min

- No gelation occurs.

**Table 4:** Selected models applied for the evaluation of RSP release and statistical parameters.

Model	Equation	Parameters	HSp	HC	ISp	IC
Korsmeyer–Peppas	$Q=Q_{\infty }\cdot K^{n}$t	*n*	0.45	0.354	0.291	0.145
		*K*	8.87	12.693	0.515	36.522
		AIC	30.53	35.975	5.840	43.500
		R^2^	0.986	0.979	0.989	0.974
Higuchi	$Q=K_{H}\sqrt{t}$	*Kk_H_*	0.13	0.110	0.250	DNF
		AIC	47.36	51.885	0.828	
		R^2^	0.936	0.845	111.967	
Weibul model	$\frac{Q}{Q_{\infty }}=\left[1-e^{-{\left(\frac{\left(t\right)}{t_{d}}\right)}^{\beta }}\right]$	*Β*	0.005	0.005	0.004	0.779
		Td*td*	0.18	0.159	0.304	1.989
		AIC	72.35	71.862	111.967	111.076
		R^2^	0.927	0.901	0.828	0.837
1st order	$\frac{Q}{Q_{\infty }}=\left(1-e^{-k_{1}\cdot t}\right)$	K*k*	0.025	0.030	0.014	0.425
		AIC	71.080	70.828	109.631	92.884
		R^2^	0.923	0.893	0.832	0.834

HSp—Hydrogel squid pen, HC—Hydrogel cuttlebone, ISp—Injectable squid pen, IC—Injectable cuttlebone, DNF: Data not fit.

## Data Availability

Not applicable.

## References

[1] Kurita K. (2001). Controlled Functionalization of the Polysaccharide Chitin. Prog. Polym. Sci..

[2] Rinaudo M. (2006). Chitin and Chitosan: Properties and Applications. Prog. Polym. Sci..

[3] Younes I., Rinaudo M., Harding D., Sashiwa H. (2015). Marine Drugs Chitin and Chitosan Preparation from Marine Sources. Structure, Properties and Applications. Mar. Drugs.

[4] Rodríguez-Vázquez M., Vega-Ruiz B., Ramos-Zúñiga R., Saldaña-Koppel D.A., Fernando Quiñones-Olvera L., Piraino F. (2015). Chitosan and Its Potential Use as a Scaffold for Tissue Engineering in Regenerative Medicine. BioMed Res. Int..

[5] Lall A., Tamo A.K., Doench I., David L., Nunes De Oliveira P., Gorzelanny C., Osorio-Madrazo A. (2020). Nanoparticles and Colloidal Hydrogels of Chitosan-Caseinate Polyelectrolyte Complexes for Drug-Controlled Release Applications. Int. J. Mol. Sci..

[6] Peers S., Montembault A., Ladavière C. (2020). Chitosan Hydrogels for Sustained Drug Delivery. J. Control. Release.

[7] Wu D., Zhu L., Li Y., Zhang X., Xu S., Yang G., Delair T. (2020). Chitosan-Based Colloidal Polyelectrolyte Complexes for Drug Delivery: A Review. Carbohydr. Polym..

[8] Chenite A., Buschmann M., Wang D., Chaput C., Kandani N. (2001). Rheological Characterisation of Thermogelling Chitosan/Glycerol-Phosphate Solutions. Carbohydr. Polym..

[9] Rahmanian-Devin P., Rahimi V.B., Askari V.R. (2021). Thermosensitive Chitosan-β-Glycerophosphate Hydrogels as Targeted Drug Delivery Systems: An Overview on Preparation and Their Applications. Adv. Pharmacol. Pharm. Sci..

[10] Amin S., Alaa El-Din B., Zhifa S., Azam A. (2016). Injectable Gel from Squid Pen Chitosan for Bone-Tissue Engineering Applications. Front. Bioeng. Biotechnol..

[11] Ngoenkam J., Faikrua A., Yasothornsrikul S., Viyoch J. (2010). Potential of an Injectable Chitosan/Starch/β-Glycerol Phosphate Hydrogel for Sustaining Normal Chondrocyte Function. Int. J. Pharm..

[12] Tsai M.L., Chang H.W., Yu H.C., Lin Y.S., Tsai Y.D. (2011). Effect of Chitosan Characteristics and Solution Conditions on Gelation Temperatures of Chitosan/2-Glycerophosphate/Nanosilver Hydrogels. Carbohydr. Polym..

[13] Schatzberg A.F., Nemeroff C.B. (2009). The American Psychiatric Publishing Textbook of Psychopharmacology.

[14] Salarvand M., Ramezani V., Salarvand F., Darabi Z.A., Akrami M. (2021). Improvement of Drug Delivery Properties of Risperidone via Preparation of Fast Dissolution Tablet Containing Nanostructured Microparticles. Iran. J. Pharm. Res..

[15] Elena de Souza L., Eckenstaler R., Syrowatka F., Beck-Broichsitter M., Benndorf R.A., Mäder K. (2021). Has PEG-PLGA Advantages for the Delivery of Hydrophobic Drugs? Risperidone as an Example. J. Drug Deliv. Sci. Technol..

[16] Lugasi L., Grinberg I., Sabag R., Madar R., Einat H., Margel S. (2020). Molecules Proteinoid Nanocapsules as Drug Delivery System for Improving Antipsychotic Activity of Risperidone. Molecules.

[17] Bellotti E., Contarini G., Geraci F., Torrisi S.A., Piazza C., Drago F., Leggio G.M., Papaleo F., Decuzzi P. (2022). Long-Lasting Rescue of Schizophrenia-Relevant Cognitive Impairments via Risperidone-Loaded MicroPlates. Drug Deliv. Transl. Res..

[18] Chen F., Liu H., Wang B., Yang L., Cai W., Jiao Z., Yang Z., Chen Y., Quan Y., Xiang X. (2020). Physiologically Based Pharmacokinetic Modeling to Understand the Absorption of Risperidone Orodispersible Film. Front. Pharmacol..

[19] Rukmangathen R., Yallamalli I.M., Yalavarthi P.R. (2019). Formulation and Biopharmaceutical Evaluation of Risperidone-Loaded Chitosan Nanoparticles for Intranasal Delivery. Drug Dev. Ind. Pharm..

[20] Kumar M., Pathak K., Misra A. (2009). Formulation and Characterization of Nanoemulsion-Based Drug Delivery System of Risperidone. Drug Dev. Ind. Pharm..

[21] Siafaka P.I., Barmpalexis P., Lazaridou M., Papageorgiou G.Z., Koutris E., Karavas E., Kostoglou M., Bikiaris D.N. (2015). Controlled Release Formulations of Risperidone Antipsychotic Drug in Novel Aliphatic Polyester Carriers: Data Analysis and Modelling. Eur. J. Pharm. Biopharm..

[22] Silva A.C., Amaral M.H., González-Mira E., Santos D., Ferreira D. (2012). Solid Lipid Nanoparticles (SLN)—Based Hydrogels as Potential Carriers for Oral Transmucosal Delivery of Risperidone: Preparation and Characterization Studies. Colloids Surf. B Biointerfaces.

[23] Badshah A., Subhan F., Rauf K., Irfan Bukhari N., Shah K., Khan S., Ahmed Z., Khan I. (2011). Development of Controlled-Release Matrix Tablet of Risperidone: Influence of Methocel^®^- and Ethocel^®^-Based Novel Polymeric Blend on In Vitro Drug Release and Bioavailability. AAPS PharmSciTech.

[24] Silva A.C., Kumar A., Wild W., Ferreira D., Santos D., Forbes B. (2012). Long-Term Stability, Biocompatibility and Oral Delivery Potential of Risperidone-Loaded Solid Lipid Nanoparticles. Int. J. Pharm..

[25] Nanaki S., Barmpalexis P., Iatrou A., Christodoulou E., Kostoglou M., Bikiaris D. (2018). Risperidone Controlled Release Microspheres Based on Poly(Lactic Acid)-Poly(Propylene Adipate) Novel Polymer Blends Appropriate for Long Acting Injectable Formulations. Pharmaceutics.

[26] D’Souza S., Faraj J.A., Giovagnoli S., DeLuca P.P. (2014). Development of Risperidone PLGA Microspheres. J. Drug Deliv..

[27] Arrouze F., Desbrieres J., Lidrissi Hassani S., Tolaimate A. (2021). Investigation of β-Chitin Extracted from Cuttlefish: Comparison with Squid β-Chitin. Polym. Bull..

[28] Galed G., Diaz E., Goycoolea F.M., Heras A. (2008). Influence of N-Deacetylation Conditions on Chitosan Production from α-Chitin. Nat. Prod. Commun..

[29] Muzzarelli R.A.A., Rocchetti R. (1985). Determination of the Degree of Acetylation of Chitosans by First Derivative Ultraviolet Spectrophotometry. Carbohydr. Polym..

[30] Rinaudo M., Milas M., Le Dung P. (1993). Characterization of Chitosan. Influence of Ionic Strength and Degree of Acetylation on Chain Expansion. Int. J. Biol. Macromol..

[31] Zhang Y., Xue C., Xue Y., Gao R., Zhang X. (2005). Determination of the Degree of Deacetylation of Chitin and Chitosan by X-ray Powder Diffraction. Carbohydr. Res..

[32] Cuong H.N., Minh N.C., van Hoa N., Trung T.S. (2016). Preparation and Characterization of High Purity β-Chitin from Squid Pens (Loligo Chenisis). Int. J. Biol. Macromol..

[33] Abdou E.S., Nagy K.S.A., Elsabee M.Z. (2008). Extraction and Characterization of Chitin and Chitosan from Local Sources. Bioresour. Technol..

[34] Zhou H.Y., Chen X.G., Kong M., Liu C.S., Cha D.S., Kennedy J.F. (2008). Effect of Molecular Weight and Degree of Chitosan Deacetylation on the Preparation and Characteristics of Chitosan Thermosensitive Hydrogel as a Delivery System. Carbohydr. Polym..

[35] Branca C., D’Angelo G., Crupi C., Khouzami K., Rifici S., Ruello G., Wanderlingh U. (2016). Role of the OH and NH Vibrational Groups in Polysaccharide-Nanocomposite Interactions: A FTIR-ATR Study on Chitosan and Chitosan/Clay Films. Polymer.

[36] Gutiérrez M.C., García-Carvajal Z.Y., Jobbágy M., Rubio F., Yuste L., Rojo F., Ferrer M.L., del Monte F. (2007). Poly(Vinyl Alcohol) Scaffolds with Tailored Morphologies for Drug Delivery and Controlled Release. Adv. Funct. Mater..

[37] Wu I.Y., Bala S., Škalko-Basnet N., di Cagno M.P. (2019). Interpreting Non-Linear Drug Diffusion Data: Utilizing Korsmeyer-Peppas Model to Study Drug Release from Liposomes. Eur. J. Pharm. Sci..

[38] Higuchi T. (1961). Rate of Release of Medicaments from Ointment Bases Containing Drugs in Suspension. J. Pharm. Sci..

[39] Siepmann J., Peppas N.A. (2011). Higuchi Equation: Derivation, Applications, Use and Misuse. Int. J. Pharm..

[40] Papadopoulou V., Kosmidis K., Vlachou M., Macheras P. (2006). On the Use of the Weibull Function for the Discernment of Drug Release Mechanisms. Int. J. Pharm..

[41] Kamdem Tamo A., Doench I., Morales Helguera A., Hoenders D., Walther A., Madrazo A.O. (2020). Biodegradation of Crystalline Cellulose Nanofibers by Means of Enzyme Immobilized-Alginate Beads and Microparticles. Polymers.

[42] Ramasamy P., Subhapradha N., Shanmugam V., Shanmugam A. (2014). Extraction, Characterization and Antioxidant Property of Chitosan from Cuttlebone Sepia Kobiensis (Hoyle 1885). Int. J. Biol. Macromol..

[43] Shushizadeh M.R., Pour E.M., Zare A., Lashkari Z. (2015). Persian Gulf β-Chitin Extraction from Sepia Pharaonis Sp. Cuttlebone and Preparation of Its Derivatives. Bioact. Carbohydr. Diet. Fibre.

[44] Aranaz I., Alcántara A.R., Civera M.C., Arias C., Elorza B., Caballero A.H., Acosta N., Velasco H., Mecerreyes D., Antonio R. (2021). Polymers Chitosan: An Overview of Its Properties and Applications. Polymers.

[45] Kolawole O.M., Lau W.M., Khutoryanskiy V.V. (2019). Chitosan/β-Glycerophosphate In Situ Gelling Mucoadhesive Systems for Intravesical Delivery of Mitomycin-C. Int. J. Pharm..

[46] Gutiérrez M.C., Ferrer M.L., del Monte F. (2008). Ice-Templated Materials: Sophisticated Structures Exhibiting Enhanced Functionalities Obtained after Unidirectional Freezing and Ice-Segregation-Induced Self-Assembly. Chem. Mater..

[47] Ebrahimi A., Sadrjavadi K., Hajialyani M., Shokoohinia Y., Fattahi A. (2018). Preparation and Characterization of Silk Fibroin Hydrogel as Injectable Implants for Sustained Release of Risperidone. Drug Dev. Ind. Pharm..

